# Fecal Microbiome and Resistome Profiling of Healthy and Diseased Pakistani Individuals Using Next-Generation Sequencing

**DOI:** 10.3390/microorganisms9030616

**Published:** 2021-03-17

**Authors:** Ome Kalsoom Afridi, Johar Ali, Jeong Ho Chang

**Affiliations:** 1 Department of Biology Education, Kyungpook National University, 80 Daehak-ro, Buk-gu, Daegu 41566, Korea; ummeafridi@gmail.com; 2 Center for Genome Sciences, Rehman Medical College, Phase-V, Hayatabad, Peshawar 25000, Pakistan; 3 Executive Development Center, Sukkur Institute of Business Administration University, Sindh 65200, Pakistan; 4 AlviArmani International, Mississauga, ON M4W-3E2, Canada; 5 Department of Biotechnology, Institute of Integrative Biosciences, CECOS University of IT & Emerging Sciences, Peshawar 25000, Pakistan

**Keywords:** antibiotics, gut microbiota, next-generation sequencing, microbial dysbiosis, loss of microbiota diversity

## Abstract

In this paper, we aimed to characterize the fecal microbiome and its resistomes of healthy and diseased subjects infected with multidrug-resistant *Escherichia coli* using next-generation sequencing (NGS). After initial screening, 26 stools samples belonging to healthy (*n* = 13) and diseased subjects (*n* = 13) were selected and subjected to NGS. A total of 23 and 42 antibiotic-resistant genes (ARGs) conferring resistance to 6 and 9 classes of antibiotics were identified in the resistomes of healthy and diseased subjects, respectively. *Bacteroidetes* were found to be the major phylum in both healthy and diseased subjects; however, *Proteobacteria* was predominantly present in the diseased subjects only. Microbial dysbiosis and predominance of various ARGs in the resistome of diseased subjects reflect the excessive usage of antibiotics in Pakistan and warrants immediate attention to regulate the use of various antimicrobials.

## 1. Introduction

The emergence of multidrug-resistant (MDR) bacteria is becoming a serious threat to human health. The human gut microbiome plays an important role in the emergence and transmission of antibiotic-resistant microbes [1]. Human gut microbiota essentially acts as a potential reservoir of antibiotic resistance genes (ARGs). A resistome includes all ARGs within a specific microbial niche [2,3]. Resistomes of different individuals are influenced by various factors such as geographical location, age, antibiotic usage, diet, environment, lifestyle, and socioeconomic status [4]. These factors also alter the composition of the human microbiome thereby making resistome closely correlated to the microbiome [3,4]. The advent of high-throughput sequencing technologies fast-tracked the microbiome research and characterized the gut microbiota as “superorganism” [5]. The human gut microbiota plays essential roles in metabolism, physiology, and development of immune system. Although gut microbiota serves as a “superorganism”, it is highly dynamic and is frequently altered by diet, age, antibiotics, various infections, and host genetic factors [6].

Among the various factors, antibiotic administration has been reported to profoundly affect the composition of the human microbiome and its resistome drastically [7]. Antibiotics administration alters the composition of the human microbiome leading to gut microbial dysbiosis [8]. The essential functions of the gut microbiome such as vitamin production, nutrient supply, and protection against pathogens are negatively affected by microbial dysbiosis [9]. A dysbiotic gut microbiome has been linked with various ailments such as developmental, immunological, and metabolic disorders. In addition, gut microbiome dysbiosis increases the susceptibility to develop various serious infectious diseases [9,10].

Antibiotics with broad-spectrum activity have been reported to affect the abundance of human gut microbiota by up to 30%, thereby causing a rapid and significant reduction in the taxonomic composition of gut microbes [11,12]. The effects of antibiotics administration on gut microbiota may last for a long time ranging from months to years. Furthermore, excessive antibiotic administration deteriorates the microenvironment of the human gut microbiome leading to the emergence of “*pathobionts”* such as *Escherichia coli*, *Salmonella enterica*, *Shigella flexneri*, *Klebsiella*, *Acinetobacter*, and *Pseudomonas* [12,13]. Consequently, the acquisition of various ARGs by these pathobionts can lead to serious health consequences [14]. Antibiotic mediated gut microbial dysbiosis has been characterized by the loss of microbial diversity, reduced abundance of specific taxa [15], increased susceptibility to various infections, escalated proliferation of various superbugs (methicillin-resistant *Staphylococcus aureus* (MRSA) and vancomycin-resistant *Enterococcus*) [16], and disruption of intestinal mucus layers [14]. Currently, a number of studies demonstrated that antibiotic mediated gut microbial dysbiosis can be effectively reversed through the incorporation of various strategies such as use of probiotics, probiotics synbiotics, fecal microbiota transplant (FMT), bacterial consortium transplant (BCT), and phage therapy [17,18]. 

Exploration of human resistome can help in designing effective diagnostic and therapeutic strategies that are the need of the hour owing to the pandemic status of AMR. Policies regulating antibiotic usage in humans and animals have been reported to influence the prevalence of ARGs in the resistomes of various individuals from different countries [19,20]. Metagenomics studies contributed significantly to understanding the bacterial communities associated ARGs in diverse samples such as human stools samples, animal fecal samples, ready-to-eat food [21], and urban resistome [22]. Large-scale metagenomic studies [19,20] explored the gut resistome of healthy people from different countries and studied the abundance pattern of ARGs. Increased abundance of ARGs was found in the gut resistome of individuals from the countries with higher antibiotic usage such as China and Spain than people belonging to the countries with strict AMR regulating policies such as Denmark. Currently, among low- to middle-income countries, Pakistan constitutes the third highest consumer of antibiotics after India and China [23]. 

Identifying the key features of country specific resistome can help understanding the abundance pattern of particular ARGs and designing interventory strategies. Large scale metagenomics studies aiming to explore the microbiome and resistome of healthy Pakistani people are scarce, and to the best of our knowledge the poultry gut microbial and abundance profile of various ARGs are only explored so far by one of our previous studies [24]. 

Strict national policies regulating the antimicrobial usage (AMU) in both human and veterinary medicine are lacking in Pakistan [23,25,26]. Higher AMU in Pakistan warrants investigating the gut resistomes of healthy and sick individuals infected with AMR bacteria in order to ascertain the prevalence of various ARGs; however, such metagenomics studies are needed in Pakistan. This cross-sectional metagenomic study aimed to investigate for the first time the fecal microbiome and its resistomes of healthy and individuals infected with AMR bacteria belonging to the Peshawar, Khyber Pakhtunkhwa region of Pakistan using next-generation sequencing (NGS).

## 2. Materials and Methods

### 2.1. Study Design

This cross-sectional study was carried out at the tertiary healthcare center of Peshawar, Pakistan (34°1′33.3012″ N and 71°33′36.4860″ E). Ethical approval (Ref: RMI/RMI-REC/Approval/33) for this study was obtained from the Ethics Committee of Tertiary Healthcare Center, Peshawar Pakistan.

### 2.2. Sample Collection

A tertiary health care center of Peshawar, Pakistan was selected for the sample collection. The study objectives were explained to the patients and informed consents were signed. Initially, we screened inpatients (*n* = 420) diagnosed with various bacterial infections. Inpatients with the confirmed bacterial infection and willingness to participate in this study were enrolled for further analysis. All the important clinical and demographic data were carefully recorded from the selected patients and healthy controls (Tables S1 and S2, Supplementary Materials). Stool samples were collected from all enrolled patients and screened for MDR bacterial strains. Stool samples under aseptic measures were streaked onto the MacConkey and Eosin Methylene Blue agar plates (Oxoid, Basingstoke, Hampshire, UK). The streaked plates were incubated for 18–24 h at 35–37 °C. Slow-growing strains were incubated for a longer duration extending up to 48 h. All the bacterial isolates were identified using the standard morphological and biochemical tests [27]. Antibiotic susceptibility testing (AST) was performed using the Kirby–Bauer disc diffusion method. Bacterial strains were tested against aminoglycosides, neomycin, gentamycin, streptomycin, chloramphenicol, quinolones and fluoroquinolones, ofloxacin, nalidixic acid, sulfonamides, sulfamethoxazole, tetracycline, beta-lactam, ampicillin, nitrofurans, and cephalosporins following Clinical and Laboratory Standards Institute (CLSI) guidelines [28]. Patients testing negative for MDR bacterial strains were excluded from this study. Stool samples from the 13 healthy participants (7 males, 6 females; mean age 48.6 ± 11 years) with no antibiotic usage in the last six months were used as negative controls for fecal microbiome and its resistome profiling.

### 2.3. Extraction, Quantification and Normalization of Genomic DNA

Genomic DNA was isolated from a 0.2 g stool sample using a commercial kit (PureLink^TM^ Microbiome DNA Purification Kit) following the manufacturer’s instructions (Invitrogen, Thermo Fisher Scientific, Waltham, MA, USA) with little modifications. The use of standard bead-beater recommended by the manufacturer was replaced by simple laboratory benchtop vortexer (CLASSIC Vortex Mixer product code F202A0173, VELP Scientifica, Via Stazione 16-20865-Usmate Velate (MB), Italy). Bead-tubes containing the samples were fixed horizontally on the pad of bench top vortexer with the help of scotch tape at room temperature and vortexed at 2000 rpm for 8 min. To assure the NGS quality control (QC), all the extracted DNA samples were then quantified using Qubit fluorometer following manufacturer’s instructions (Qubit™ fluorometer, Invitrogen, Carlsbad, CA, USA) [29]. The quality of DNA samples were also checked using 1.0% agarose gel. Following quality control (DNA qualitative and quantitative analysis), all the DNA samples were then normalized to 0.2 ng/µL (1 ng/5µL).

### 2.4. NGS Libraries Preparation

Sequencing libraries were prepared using Illumina^®^ (San Diego, CA, USA) Nextera XT DNA Library Preparation Kit (FC-131-1096) and Nextera XT Index Kit v2 Set A (FC-131-2001) as per the manufacturer’s instructions. For library preparation, 1ng dsDNA was subjected to tagmentation. dsDNA was fragmented and adapters were added to both ends. Unique Illumina idexes (i7/i5) were added to tagmented DNA through limited cycles of PCR amplification. The PCR amplified products were purified using AMPure XP beads and washed twice with 80% freshly prepared ethanol. Then, bead-based normalization of the purified products were done by using 45µL mixture of beads Library Normalization Additives 1 (LNA1), and Library Normalization Beads 1 (LNB1), and washed by using wash solution provided in the kit. Finally, each library was eluted in 0.1N NaOH and stored in storage buffer. All the libraries were pooled by mixing 5 µL each and subsequently 24 µL from the pooled sample (pooled DNA libraries) was mixed with 576 µL HTI (Hybridization Buffer) and subjected to denaturation at 98 °C for 2 min. Furthermore, 30 µL of 12.5 pM PhiX (internal sequencing control) was added to the pooled libraries thereby making a final volume of 600 µL, which was then loaded on to Illumina MiSeq Reagent Kit v2 (300 cycles; MS-103-1002, Illumina Inc., CA, USA) for paired end sequencing (2 × 150 bp) on Illumina MiSeq sequencer (Illumina, San Diego, CA, USA).

### 2.5. NGS Bioinformatics Analysis

After FASTQ files (raw data) were generated through MiSeq (Illumina sequencer), 26 dual-index barcoded metagenomes were demultiplexed using CASAVA 1.8.2. The sorted FASTQ files were then subjected to NGS QC using Trimmomatic 0.36 to remove all technical biases, low-quality reads (Q > 30), and adapters. To assure shotgun metagenome sequencing QC, filtered FASTQ files were further filtered from the host DNA using a computational tool KneadData v. 0.6. The filtered, high quality metagenomics data sets (*n* = 26) were subjected to unique clade-specific marker genes based microbial profiling using a computational tool Metagenomic Phylogenetic Analysis (MetaPhlAn3) [30]. Bacterial taxonomic profiling was followed by resistome analysis. All the high quality filtered reads were subjected to the resistome analysis using the default settings of Bowtie2 through Antimicrobial Resistance Identification By Assembly (ARIBA) MEGAres database [31]. All ARGs were confirmed through a minimum of 16 reads (2 × 150 bp) each and a 100% identity match to the reference genome. All read alignments were manually inspected to validate the existence of various ARGs. The abundance of ARGs were estimated on the basis of relative number of reads assigned to each ARG.

## 3. Results

### 3.1. Selection of Samples for Shotgun Metagenome Analysis

Out of 420 screened patients, 13 patients (7 males, 6 females) with a mean age of 48.6 ± 11 years were found resistant to more than 3 major classes of antibiotics and were named as MDR *E. coli* infected patients. To explore the gut microbiome and resistome of MDR *E. coli* infected patients (hereafter named as diseased subjects) in addition to the healthy controls, we selected the 13 MDR infected stool samples for shotgun metagenome sequencing. A total of 26 stool samples belonging to healthy and diseased subjects (13 each) were analyzed using shotgun metagenome sequencing. 

### 3.2. Shotgun Metagenome Sequencing

The NGS quality filtering system discarded reads quality score <Q30 and read length less than 60 nucleotides. A total of 28,842,214 filtered paired-end reads were obtained. The high-quality NGS reads (comprising of healthy and diseased samples) were processed for bacterial taxonomic and resistome profiling.

### 3.3. Bacterial Profiling of Fecal Microbiome of Healthy Subjects at Various Levels

**Figure 1:**
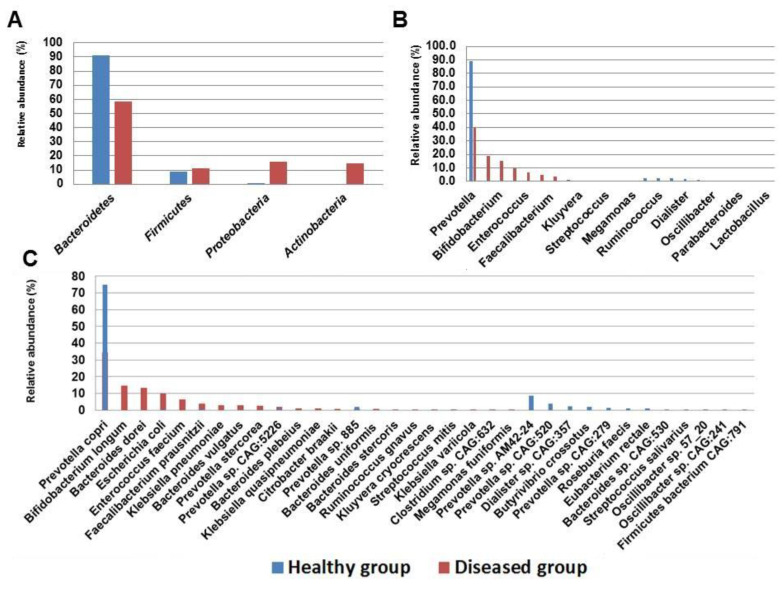
Taxonomic profiling of fecal microbiome in healthy and diseased subjects at various levels. Relative abundance of fecal microflora of healthy and diseased subjects at phylum level (**A**), genus level (**B**), and species level (**C**).

The percentage abundance of different taxonomic ranks was quantified based on reads assigned to each clade. A total of 3 phyla, 6 classes, 6 orders, 6 families, and 9 genera. Diverse species were identified in the fecal microbiome of healthy subjects (Figure 1), among which 15 were dominantly found. *Bacteroidetes* were identified as the major representative phylum with the relative abundance of 90.8% followed by *Firmicutes* (9%). *Proteobacteria* was identified as the minor phylum with the relative abundance of <1% (Figure 1A). The major class identified was *Bacteroidia* (90.8%), followed by Clostridia (6.6%), and *Negativicutes* (1.9%). While the minor (<1%) classes identified *Bacilli*, *Firmicutes* unclassified, and *Gammaproteobacteria*. Among the total 6 orders identified, *Bacteroidales* (90.8%) were identified as the major one followed by *Clostridiales* (6.6%), and *Veillonellales* (1.9%). While the minor orders (<1%) identified were *Lactobacillales*, *Firmicutes* unclassified, and *Enterobacterales. Prevotellaceae* (90.3%) was identified as the dominant family followed by *Lachnospiraceae* (5.7%), and *Veillonellaceae* (1.9%). The minor (<1%) families identified were *Enterobacteriaceae*, *Ruminococcaceae*, and *Bacteroidaceae*. *Prevotella* was identified as the major genus with the relative abundance of 90.3%. Genus *Prevotella* was followed by *Roseburia* (2.2%), *Butyrivibrio* (2.1%), *Dialister* (1.8%), unclassified *Lachnospiraceae* (1.1%). The minor genera (<1%) were found to be *Bacteroides*, *Faecalibacterium*, *Escherichia*, and *Klebsiella* (Figure 1B)*. Prevotella copri* was identified as the major species (74.6%). A number of other *Prevotella* species were also dominantly found, for instance *Prevotella* sp. *AM42 24* (8.2%), *Prevotella* sp. *CAG 520* (3.6%), *Prevotella* sp. *885* (1.7%), and *Prevotella* sp. *CAG 279* (1.3%). Similarly, dominant species other than *Prevotella* spp., are *Butyrivibrio crossotus* (2.1%), *Dialister* sp. *CAG 357* (1.8%), *Roseburia* faecis (1.3%), *Eubacterium rectale* (1.1%). Diverse minor species (<1%) were identified (Figure 1C), for instance, *E. coli*, *Klebsiella pneumoniae*, *Prevotella stercorea*, *Prevotella* sp. *CAG 5226*, and *Prevotella* *stercorea.*

### 3.4. Bacterial Profiling of Fecal Microbiome of Diseased Subjects at Various Levels

A total of 4 phyla, 6 classes, 6 orders, 10 families, 13 genera, and 23 species were identified in the fecal microbiome of diseased subjects (Table 1, Figure 1). *Bacteroidetes* (58.3%) was identified as the major phylum followed by *Proteobacteria* (15.8%), *Actinobacteria* (14.7%), and *Firmicutes* (11.2%) (Figure 1A). *Bacteroidia* (58.3%) was identified as the major class followed by *Gammaproteobacteria* (15.8%), *Actinobacteria* (14.7%), *Bacilli* (6.7%), and *Clostridia (*4.3%), while minor (<1%) class identified was *Negativicutes*. *Bacteroidales* was identified as the dominant order followed by *Enterobacterales* (15.8%), *Bifidobacteriales* (14.7%), *Lactobacillales* (6.7%), and *Clostridiales* (4.3%), while *Selenomonadales* was identified as the minor (<1%) order. *Prevotellaceae* 39.6% was identified as the major family followed by *Bacteroidaceae* 18.7%, *Enterobacteriaceae* 15.8%, *Bifidobacteriaceae* 14.7%, *Enterococcaceae* 6.3%, and *Ruminococcaceae* (3.8%). The minor (<1%) families identified were *Lachnospiraceae*, *Streptococcaceae*, *Clostridiaceae*, and *Selenomonadaceae. Prevotella* (39.6%) was identified as the major genus followed by *Bacteroides* (18.7%), *Bifidobacterium* (14.7%), *Escherichia* (9.6%), *Enterococci* (6.3%), *Klebsiella* (4.9%), and *Faecalibacterium* *(*3.8%). The minor (<1%) genera identified were *Streptococcus*, *Clostridium*, *Blautia*, *Megamonas*, *Citrobacter*, and *Kluyvera* (Figure 1B). The species identified were *Prevotella copri* (34.5%) *Bifidobacterium longum* (14.7%), *Bacteroides dorei* (13.3%), *E. coli* (9.5%), *Enterococcus faecium* (6.3%), *Faecalibacterium prausnitzii* (3.8%), *Klebsiella pneumoniae* (3%), *Bacteroides* vulgatus (2.8%), *Prevotella stercorea* (2.6%), *Prevotella* sp. *CAG 5226* (*1.5*%), *Klebsiella quasipneumoniae* (1.4%), and *Bacteroides plebeius* (1.2%). Furthermore, 11 minor (<1%) species were also identified in the fecal microbiome of diseased subjects (Figure 1C).

### 3.5. Comparative Bacterial Profiling of Healthy and Diseased Subjects

*Bacteroidetes* were identified as a major abundant phylum in both healthy controls (90.8%) and diseased subjects (58.3%). Albeit, *Bacteroidetes* constitute the major abundant phylum in both healthy and diseased subjects, however, relative percentage abundance of *Bacteroidetes* was found to be higher in healthy controls (Figure 1, Table 1). *Firmicutes* (8.9%) constitute as the second abundant phylum in healthy controls while *Proteobacteria* (15.8%) was the second major phylum in the diseased subjects. Conversely, *Proteobacteria* was found to be the least (<1%) abundant phylum in the healthy controls. The percentage abundance of microbial communities at different taxonomic ranks such as phylum, class, order, family, genus, and species of both healthy and diseased subjects are shown in Figure 1A–C. *Prevotella* was identified as the major genus in both healthy controls (90.3%) and diseased subjects (39.6%); however, the relative percentage abundances of both groups vary greatly (Figure 1C, Table 1). Furthermore, the various pathobionts genera such as *Escherichia* and *Klebsiella* were abundantly found in the fecal microbiome of diseased subjects, while in the healthy controls these genera constituted for <1%.

### 3.6. NGS-Based Resistome Analysis

Resistome analysis of healthy and diseased subjects revealed the presence of diverse ARGs (355,959 reads) conferring resistance to multiple antibiotics; namely, tetracycline, beta-lactam, macrolide-lincosamide-streptogramin (MLS), aminoglycoside, sulphonamide, multidrug efflux pump system, rifampin, quinolone, and trimethoprim. 

### 3.7. Resistome of Healthy Subjects

**Figure 2:**
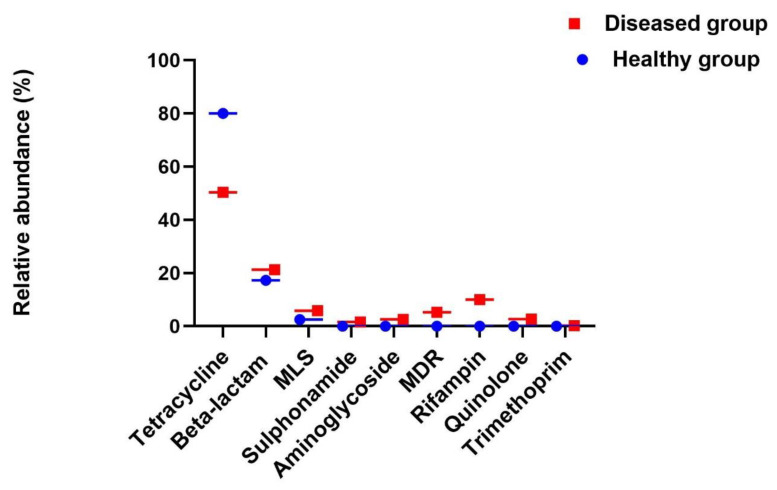
The relative abundance of various antibiotic resistance gene (ARG) types identified in the resistomes of healthy and diseased subjects. The relative abundance of each ARG type was estimated on the basis of total number of reads. MLS—macrolide-lincosamide-streptogramin; MDR—multidrug efflux pump.

The resistome of healthy controls were composed of 23 ARGs conferring resistance to various classes of antibiotics such as tetracycline, beta-lactam, MLS, sulphonamide, aminoglycoside, and multidrug efflux pumps. The abundance of various ARGs were estimated on the basis of their reads for instance, tetracycline associated ARGs (80%; *n* = 7, *tet*32, *tet*40, *tet*A) were found to be the major genes followed by beta-lactam (17.3%; *n* = 5; *blacfx*A3, *blacfx*A6, *bla*CTX), and MLS (2.5%; *n* = 3, *Mph*A, *erm*F, *erm*B). Tetracycline, beta-lactam, and MLS associated ARGs were found as the dominant genes while the relative abundance of ARGs associated with efflux pump (*n* = 3, *mdt*A, *emr*K, *mdt*L), aminoglycoside (*n* = 3, *aad*A5, *aph*(3′′)-Ib, *aac*A4), sulphonamide (*n* = 2, *sul*1/*sul*3) were found to be < 1% (Figure 2, Table 2).

### 3.8. Resistome of Diseased Subjects

The resistome of diseased subjects were composed of 42 ARGs conferring resistance to 9 classes of antibiotics such as tetracycline, beta-lactam, rifampin, MLS, multidrug efflux pumps, quinolone, aminoglycoside, sulphonamide, and trimethoprim (Figure 2, Table 2). Tetracycline associated ARGs were found to be the major genes (50.4%; *n* = 8, *tet*M, *tet*O, *tet*Q), followed by beta-lactam (21.3%; *n* = 8, *bla*CTX-M, *bla*CMH-1, *bla*CMY), rifampin (10%; *n* = 1, *rpo*B), MLS (5.9%; *n* = 5, *erm*B, *erm*F, *erm*X), multidrug efflux pump (5.3%, *n* = 10 *mdt*G, *mdt*H, *mdt*N), quinolone (2.7%; *n* = 2, *qnr*B, *qnr*S), aminoglycoside (2.6%; *n* = 5, *aac*6, *aad*A5, *acr*E), sulphonamide (1.6%; *n* = 2 *sul*1, *sul*2), and trimethoprim (0.2%; *n* = 1, *Dfr*A17).

### 3.9. Comparative Resistome Analysis

A greater number of ARGs were identified in diseased subjects (*n* = 42) than that of healthy controls (*n* = 23). Similarly, in contrast to the healthy subjects, the resistome of diseased subjects showed higher diversity in terms of ARGs to be associated with 9 classes of antibiotics namely tetracycline, beta-lactam, macrolide-lincosamide-streptogramin (MLS), aminoglycoside, sulphonamide, multidrug efflux pump system, rifampin, quinolone, and trimethoprim (Figure 2). While resistome of healthy controls showed resistance to only 6 classes of antibiotics namely tetracycline, beta-lactam, MLS, sulphonamide, aminoglycoside, and multidrug efflux pumps (Table 2). Moreover, tetracycline associated ARGs were found abundantly in the gut resistomes of both healthy (80%) and diseased (50.4%) subjects. Similarly, beta-lactam-associated ARGs were found to be the second most abundant ARGs in the gut resistomes of both healthy and diseased subjects with a relative abundance of 17.3% and 21.3%, respectively (Figure 2).

## 4. Discussion

Shotgun metagenome sequencing has been widely used to explore various ARGs in diverse hosts [14,19,20]. NGS-based metagenomic approach is widely adopted by the developed countries for resistome analysis for surveillance and diagnostic purposes however, being a third-world country with limited resources, Pakistan still lags behind in this race [32,33]. The present study is the first of its kind, which used an NGS-based metagenomic approach to investigate the gut resistome of healthy and diseased human subjects belonging to Peshawar, Pakistan. A number of ARGs were identified in the resistome of both healthy and diseased subjects. The presence of various ARG types in the resistome of healthy subjects is in agreement with a recent metagenomic study [34]. A metagenomic study identified various ARGs in the resistome of Yanomami Amerindian villagers whose ancestors were isolated for more than 11,000 years and have never been administered any synthetic antibiotics [35]. The presence of ARGs in these isolated villagers implies that ARGs are inherent features of the human microbiome. Although ARGs have been implicated as the inherent features of the human microbiome, albeit excessive antibiotic administration upsurges the acquisition, transmission, and dissemination of ARGs in the gut resistomes of different hosts [36]. Moreover, the antibiotic treatment causes the modification of gut resistome thereby leading to the predominance of resistant strains and their associated ARGs [37,38]. In Pakistan, antibiotics are available over the counter and self-medication is commonly practiced in the general population and also in hospital community settings [39]. A recent multicenter cross-sectional study carried out in urban areas of Punjab, Pakistan, evaluated the sale extent of non-prescribed antibiotics. Out of 353 pharmacy stores, 96.9% of medical stores and pharmacy centers were found to be dispensing antibiotics without any prescription where as 3.1% of pharmacy stores were found to be dispensing antibiotics on the basis of authentic prescription [40]. Recently, the total consumption of antibiotics in Pakistan has been increased by 65% from the year 2000 to 2010 [41]. Furthermore, a recent study also highlighted that the inappropriate use of antibiotics in both general and hospital community settings contributes to the deadly AMR situation in Pakistan [42]. We, therefore, speculate that widespread presence of diverse ARGs in the resistomes of healthy subjects is likely due to the unjustified use of antibiotics in Pakistan. 

Greater diversity and predominance of ARGs were observed in the resistome of diseased subjects, which could be justified by the fact that they were already on antibiotic treatment for various bacterial infections. The association of antibiotic administration to the emergence of increased ARGs in the diseased subjects is supported by a previous study indicating that intake of antimicrobials causes potential expansion of gut resistome [43]. A number of studies indicated the widespread presence of various AMR bacterial strains and their associated ARGs, for instance, a study carried out in tertiary health care hospital of indicated that all *Acinetobacter* and greater than 70% *P. aeruginosa* isolates were found to be MDR [44,45]. Furthermore, our results are supported by a prior study that explored the publicly available datasets of 24 metagenomic samples collected from the healthy controls (*n* = 6) and antibiotic-treated individuals (*n* = 18). Their results indicate that antibiotic administration in diseased subjects for a short duration causes the diversification of gut resistome, increases the abundance of ARGs and specific pathogenic bacterial strains [46]. Moreover, using shotgun metagenomics, a group of researchers quantified alterations in the gut microbiota of hematological patients under antibiotic prophylaxis grouped in two different cohorts. Ciprofloxacin was administered in one cohort while cotrimoxazole in the other. Their results indicated reduction in gut microbial diversity of both the treated cohorts up to the similar extent, however their gut resistome differs owing to the use of a particular group of antibiotics [1]. In addition, the higher diversity of ARGs in diseased subjects is in agreement with a recent study indicating that the overall relative abundance of various ARGs was higher significantly in patients than that of healthy controls [34]. We observed a high abundance of tetracycline and beta-lactam associated ARGs in the resistomes of both healthy and diseased subjects. The prevalence of tetracycline associated ARGs in the healthy subjects is consistent with a previous study [47]. Feng et al. [47] established a catalog of resistome to study the abundance pattern of various ARG types and fecal microbial communities from healthy people (*n* = 180) belonging to 11 different countries. They identified 507 subtypes (from 20 types of ARGs), among which the tetracycline associated ARGs have been grouped in the top seven prevalent ARGs. Their results implies that tetracycline associated ARGs occurs commonly in human resistome [47]. Globally, tetracycline associated ARGs predominantly present in the human gut resistomes as evidenced by few studies indicating the high abundance of tetracycline associated ARGs among all the identified ARG types in the resistomes of Chinese, Danish, American, and Spanish people [20,48].

Data related to metagenomic studies are scarce in Pakistan; however, the drug resistance pattern of *Shigella* isolates belonging to the Faisalabad region was studied using a molecular approach. *Shigella* isolates exhibited resistance predominantly to ampicillin (96.84%) and tetracycline (93.68%) which is in line with our results indicating a high abundance of tetracycline and beta-lactam associated ARGs in the diseased subjects [49]. The high diversity of various ARGs conferring resistance to multiple classes of antibiotics (Table 2) in the diseased subjects can be attributed to the self-medication, over-the-counter availability of antibiotics, wrong prescription practices, and lack of legislation regulating antibiotic usage across Pakistan [50]. Furthermore, physicians in Pakistan usually prescribe antibiotics to the patients prior to the result of AST which could be another possible reason for the diversification of their gut resistomes. Prescription of antibiotics without AST has been considered as the potential cause of AMR in the developing countries such as India and Pakistan [51,52]. The widespread inappropriate use of antibiotics in Pakistan has led to the emergence of ARGs not only in human but also in food animals and their surrounding environments such as water bodies. In addition to humans, various ARGs have been detected in various environmental samples of Pakistan. Using real-time PCR, a research group detected the high abundance of *sulI* and *dfrA1* in the river bodies of Northern Pakistan [53].

The increased abundance of *Bacteroidetes* in the fecal microbiota of healthy controls is supported by the fact that *Bacteroidetes* are the major representatives of the normal gut [54]. *Bacteroidetes* were identified as the most abundant phylum in both healthy and diseased subjects; however, their relative abundances varied greatly (Figure 1, Table 1). Moreover, *Proteobacteria* was identified as the second major phyla in the diseased subjects while in the healthy controls it accounted for <1% which implies that the lower diversity of microbial communities was likely caused by microbial dysbiosis in diseased subjects. The predominance of *Proteobacteria* in the diseased subjects is in agreement with previous studies indicating its association with different diseases and microbial disruption [55,56,57]. Disruption of gut microbiota with antibiotic usage is a well-established fact [56,57]. Different antibiotics cause gut microbial dysbiosis in mice; for instance, mice treated with aztreonam and metronidazole cause a reduction in the relative abundance of Bacteroidetes while the growth of *Klebsiella* sp., *Helicobacter* sp., and *Ruminococcus* sp. were found to be enhanced [58].

Dietary interventions also significantly alter the composition of gut microbiota [59]. Pakistani diet is mainly omnivorous type; however, during various ailments people usually prefer to consume soft fermented foods in combination with other food items (Table S1, Supplementary Materials). The diseased subjects in our study changed their diet from an omnivorous type to softer and/or fermented items composed of natural homemade yoghurt, boiled rice with lentils, and custard. Compared to the healthy subjects, the high abundance of *Actinobacteria* in the diseased subjects could be justified by their dietary interventions (especially intake of natural yoghurt) which is in line with the previous studies indicating the prevalence of *Bifidobacterium* in patients receiving yoghurt [60,61,62]. 

Overall, a greater microbial diversity was observed in healthy controls than that of diseased subjects which are in agreement with the previous studies [34,47,55,56]. At the genus level, a high abundance of *Prevotella* was observed in the healthy controls than that of diseased subjects. The predominance of *Prevotella* in both healthy and diseased subjects can be attributed to the Pakistani diet which is mostly plant-based [63]. However, we speculate that antibiotic administration in diseased subjects reduced the abundance of *Prevotella* thereby leading to the expansion of pathobiontic genera such as *Klebsiella* and *Escherichia*. Moreover, a higher abundance of important pathobiontic species such as *E. coli*, *Klebsiella pneumonia*, and *Klebsiella quasipneumoniae* was observed in diseased subjects while the healthy controls harbored a high proportion of several beneficial bacteria (Table 1). The high abundance of pathobionts in the diseased subjects is in line with a previous study [34]. The various pathogenic species such as *E. coli*, *Klebsiella pneumonia*, and *Klebsiella quasipneumoniae* has been considered as the signatures of gut microbial dysbiosis [57]. The relative abundance of *E. coli*, *Klebsiella* spp. are normally low however, these species have the potential to expand and dominates in the intestines during microbial dysbiosis thereby leading to serious health consequences [64].

## 5. Conclusions and Future Perspective

The present study investigated the fecal microbiome and resistome of healthy and diseased Pakistani individuals. Higher microbial diversity was observed in the fecal microbiome of healthy controls than that of diseased subjects. A high abundance of ARGs conferring resistance to multiple antibiotics was observed in the resistome of diseased subjects than that of healthy individuals. Microbial dysbiosis and predominance of various ARGs in the resistome of diseased subjects reflect the excessive usage of antibiotics in Pakistan and warrants immediate attention to regulate the use of various antimicrobials. Using a high-throughput shotgun metagenome sequencing, the present study is the first of its kind which characterized the fecal microbiome and resistome of both healthy and diseased Pakistani subjects. This study showed that how a healthy gut microbiome and its resistome differs from the diseased one. Furthermore, this study also highlighted how antibiotic misuse perpetuates the structure of gut microbial communities leading to a dysbiotic flora.

Identifying the pathogenic bacterial strains and their associated ARGs can help to design novel precision medicine-based therapeutic strategies for targeted mitigation of resistant microbes. Currently, microbial strategies used for targeted mitigation of resistant microbes are either based on bacteriophages or microbial remediation [65]. Bacteriophages have been considered as the potential weapon against MDR, extensively drug-resistant (XDR), and pan drug-resistant (PDR) in developed counties [66]. A group of researchers used a cocktail of different members of wild phages to successfully treat a wound infection caused by MDR *A. baumannii* [67]. Similarly, compared to the conventional antibiotic therapy, FMT profoundly improved the treatment outcomes of *Clostridioides difficile* infection [68]. Currently, in the USA and Canada, FMT has been considered as a biological medicine (biologic drug) [69].

With continued misuse of antibiotics, gut microbial diversity will be reduced leading to the severe absence of useful bacteria thereby causing grave future health issues. Going forward, strict legislation with proper implementation of antibiotic usage is required. Here, we propose the establishment of a biobank of useful microbes ready to be used as a supplement in case it disappeared from the gut microbial ecosystem. In the future, a detailed study with a large sample size is required to create a comprehensive library of all the microbes from different host species for subsequent use and reference backed up by a comprehensive biobank of the identified microbes.

## Figures and Tables

**Table 1:** Bacterial profiling fecal microbiota of healthy and diseased subjects were calculated using Metagenomic Phylogenetic Analysis (MetaPhlAn3).

Taxonomic Rank		Control Group (%)	Diseased Group (%)	SD±
**Phylum**	*Bacteroidetes*	90.8	58.3	23
	*Firmicutes*	9	11.2	1.6
	*Proteobacteria*	0.2	15.8	11.1
	*Actinobacteria*	NA	14.7	NA
**Family**	*Bacteroidaceae*	0.4	18.7	12.9
	*Prevotellaceae*	90.3	39.6	35.8
	*Lachnospiraceae*	5.7	0.4	3.7
	*Ruminococcaceae*	0.5	3.8	2.3
	*Veillonellaceae*	1.9	NA	NA
	*Enterobacteriaceae*	0.2	15.8	11.1
	*Bifidobacteriaceae*	NA	14.7	NA
	*Enterococcaceae*	NA	6.3	NA
**Genus**	*Bacteroides*	0.4	18.7	12.9
	*Prevotella*	90.3	39.6	35.8
	*Butyrivibrio*	2.1	NA	NA
	*Lachnospiraceae unclassified*	1.1	NA	NA
	*Roseburia*	2.2	NA	NA
	*Faecalibacterium*	0.5	3.8	2.4
	*Dialister*	1.8	NA	NA
	*Escherichia*	0.1	9.6	6.7
	*Klebsiella*	0.05	4.9	3.5
	*Bifidobacterium*	NA	14.7	NA
	*Enterococcus*	NA	6.3	NA
**Species**	*Bacteroides vulgatus*	0.1	2.9	1.9
	*Prevotella copri*	74.6	34.5	28.4
	*Prevotella* sp. *885*	1.7	0.9	0.6
	*Prevotella* sp. *AM42 24*	8.2	NA	NA
	*Prevotella* sp. *CAG 279*	1.3	NA	NA
	*Prevotella* sp. *CAG 520*	3.6	NA	NA
	*Prevotella* sp. *CAG 5226*	0.7	1.6	0.6
	*Prevotella stercorea*	0.2	2.6	1.8
	*Butyrivibrio crossotus*	2.1	NA	NA
	*Eubacterium rectale*	1.1	NA	NA
	*Roseburia faecis*	1.3	NA	NA
	*Faecalibacterium prausnitzii*	0.53	3.8	2.4
	*Dialister* sp. *CAG 357*	1.8	NA	NA
	*E. coli*	0.1	9.6	6.7
	*Klebsiella pneumoniae*	0.05	3.1	2.1
	*Bifidobacterium longum*	NA	14.7	NA
	*Bacteroides dorei*	NA	13.3	NA
	*Bacteroides plebeius*	NA	1.2	NA
	*Enterococcus faecium*	NA	6.3	NA
	*Klebsiella quasipneumoniae*	NA	1.5	NA

NA—not applicable (absence of particular taxonomic rank or parameter).

**Table 2:** Diversity of various antibiotic resistance genes (ARGs) in the gut resistome of healthy and diseased subjects.

ARG Type	Resistome of Healthy Subjects	Resistome of Diseased Subjects
Tetracycline	*tet*32, *tet*40, *tet*A, *tet*O, *tet*Q, *tet*R, *tet*W	*tet*M, *tet*O, *tet*Q, *tet*S, *tet*W, *tet*A, *tet*B, *tet*R
Beta-lactam	*aci*1, *blacfx*A3, *blacfx*A6, *bla*CTX, *bla*TEM	*bla*CTX*-*M, *bla*CMH-1, *bla*CMY, *bla*cfxA3, *bla*cfxA6, *bla*NDM-1, *bla*OXY-1, *bla*TEM-1
MLS ^1^	*Mph*A, *erm*F, *erm*B	*erm*B, *erm*F, *erm*X, *mef*A, *Msr*D
Sulphonamide	*sul*1, *sul*3	*sul*1, *sul*2
Aminoglycoside	*aad*A5, *aph(3”)-Ib*, *aac*A4	*aac*6, *aadA*5, *acr*E, *acr*F, *aph*3
MDR ^2^	*mdt*A, *emr*K, *mdt*L	*mdt*G, *mdt*H, *mdt*N, *mdt*F, *mdt*C, *mdt*O, *msb*A, *adeC*, *emr*K, *emr*R

^1^ MLS—macrolide-lincosamide-streptogramin, ^2^ MDR—multidrug efflux pump system.

## Data Availability

Raw metagenomic data generated in this study has been deposited to the publicly accessible NCBI Sequence Read Archive (SRA) under the accession number: PRJNA612780.

## Supplementary Materials

The following are available online at https://www.mdpi.com/2076-2607/9/3/616/s1, Table S1: Details of Pakistani *omnivorous diet consumed by healthy participants and soft fermented diet received by diseased subjects*, Table S2: Demographic and clinical details of healthy and diseased subjects.
