# Supplementary material for: Fecal Microbiome and Resistome Profiling of Healthy and Diseased Pakistani Individuals Using Next-Generation Sequencing
Source: Microorganisms. 2021 Mar 17;9(3):616. doi: 10.3390/microorganisms9030616 (PMC8002588; doi:10.3390/microorganisms9030616)
Supplement: Supplementary file 1 [file microorganisms-09-00616-s001.pdf]

microorganisms

Supplementary Table S1. Details of Pakistani o*mnivorous* diet consumed by healthy participants and soft fermented diet received by diseased subjects

Omnivorous diet	**Soft fermented diet**
Paratha/chapatti/naan<br>Fried eggs/ *omelette*<br>Kebab (chopped beef with spices and different grains)<br>Chicken curry<br>Vegetable curry<br>Vegetable curry mixed with meat<br>Dhal (pulses of different types)<br>Chicken curry<br>Meat karahi<br>Rice (different types)<br>Black tea (Chai)<br>Samosas (pastry fried deeply)<br>Keema Curry (chopped meat)<br>Fast food<br>Fresh vegetables salad	Soft white Bread<br>*Dahi* (traditional natural yogurt)<br>Kitchari (made up of soft boiled rice, lentils or split peas, spices, and oil)<br>Chicken soup<br>Custard

Supplementary Table S2. Demographic and clinical details of healthy and diseased subjects

**Variables**	**Healthy group**	**Diseased group**
Mean age	48.6 ± 11	48.6 ± 11
Geographical designation	South Asian	South Asian
Nationality	Pakistani	Pakistani
Ethnicity	Pashtuns	Pashtuns
Residence	Peshawar (urban)	Peshawar (urban)
Socioeconomic status	Low	Low
Education	Educated (n=10)	Educated (n=04)
	Uneducated (n=03)	Uneducated (n=09)
History of self-medication	No	Yes
Antibiotic intake in last 6 months	No	Yes
Commonly used antibiotics	Not taking any	Amoxicillin
		Carbapenems
		Fourth-generation cephalosporins
		Aminoglycosides
		Fluoroquinolones
		Sulfamethoxazole and trimethoprim
		Dicloxacillin, cephalexin, and clindamycin
		Nitrofurantoin,
Infectious diseases	Not infected	Bacterial gastroenteritis caused by *Enterobacter* (n=5)
		Bacterial gastroenteritis caused by *Escherichia coli* (n=6)
		Pseudomonas aeruginosa (n=2)
